# Supplementary material for: Global high-resolution estimates of the UN Human Development Index using satellite imagery and machine learning
Source: Nat Commun. 2026 Feb 17;17:1315. doi: 10.1038/s41467-026-68805-6 (PMC12913631; doi:10.1038/s41467-026-68805-6)
Supplement: Supplementary file 1 — Supplementary Information [file 41467_2026_68805_MOESM1_ESM.pdf]

Supplementary Information for Global high-resolution estimates of the UN Human Development Index using satellite imagery and machine learning

Luke Sherman, Jonathan Proctor*, Hannah Druckenmiller,
Heriberto Tapia, Solomon Hsiang
* Corresponding author. Email: jon.proctor@ubc.ca

This PDF file includes:

Tables S1 to S5
Figures S1 to S10
Supplementary Discussion Sections S1 to S6
Supplementary References

Supplementary tables

HDI trained at:	**Predicted at province level** (n=1,381)			
	Full variation performance		*Within-country performance*	
	ρ^2	R^2	ρ^2	R^2
	(1)	(2)	(3)	(4)
**Within-country** (n=1,363)	0.96	0.96	0.52	0.52
**Province level** (n=1,381)	0.83	0.83	0.44	0.24
**Country level** (n=145)	0.74	0.74	0.28	< 0

HDI trained at:	**Predicted at municipality level in Indonesia** (n=505)			
	Within-country performance		*Within-province performance*	
	ρ^2	R^2	ρ^2	R^2
	(3)	(4)	(5)	(6)
**Within-country** (n=1,363)	0.62	0.61	0.53	0.53
**Province level** (n=1,381)	0.5	0.37	0.51	0.37
**Country level** (n=145)	0.35	< 0	0.36	< 0

HDI trained at:	**Predicted at municipality level in Brazil** (n=5,584)			
	Within-country performance		*Within-province performance*	
	ρ^2	R^2	ρ^2	R^2
	(3)	(4)	(5)	(6)
**Within-country** (n=1,363)	0.48	0.48	0.33	0.20
**Province level** (n=1,381)	0.46	0.36	0.31	< 0
**Country level** (n=145)	0.29	< 0	0.18	< 0

HDI trained at:	**Predicted at municipality level in Mexico** (n=2,457)			
	Within-country performance		*Within-province performance*	
	ρ^2	R^2	ρ^2	R^2
	(3)	(4)	(5)	(6)
**Within-country** (n=1,363)	0.5	0.45	0.31	0.29
**Province level** (n=1,381)	0.31	0.31	0.26	0.23
**Country level** (n=145)	0.16	< 0	0.12	< 0

IWI trained at:	**Predicted at DHS cluster level** (n=51,996)					
	Full variation performance		*Within-country performance*		*Within-province performance*	
	ρ^2	R^2	ρ^2	R^2	ρ^2	R^2
	(1)	(2)	(3)	(4)	(5)	(6)
**Within-country** (n=862)	0.71	0.67	0.56	0.56	0.36	0.36
**Province level** (n=862)	0.44	0.28	0.21	0.08	0.24	0.1
**Country level** (n=85)	0.31	< 0	0.13	< 0	0.13	< 0

NL trained only on MOSAIKS at:	**Predicted at municipality level** (n=62,536)					
	Full variation performance		*Within-country performance*		*Within-province performance*	
	ρ^2	R^2	ρ^2	R^2	ρ^2	R^2
	(1)	(2)	(3)	(4)	(5)	(6)
**Within-country** (n=2,852)	0.73	0.7	0.64	0.63	0.61	0.57
**Province level** (n=2,852)	0.65	0.61	0.57	0.45	0.53	0.35
**Country level** (n=170)	0.44	0.3	0.36	0.08	0.31	< 0

Table S1: Performance for models trained to predict HDI, IWI, and population-weighted nightlight luminosity (NL). Models for HDI and IWI use a combination of MOSAIKS and population-weighted NL features. We show performance evaluated at the province level for HDI and evaluate downscaled performance for HDI, IWI, and NL. Performance scatters from the within-country models are shown in Figure 2. All predictions are made for the year 2019.

		**Predicted at province level** (n=378)			
		Full variation performance		*Within-country performance*	
		ρ^2	R^2	ρ^2	R^2
HDI trained at:	***Features***	(1)	(2)	(3)	(4)
**Within-country**	**MOSAIKS+NL**	0.97	0.97	0.43	0.42
**Province level**	**MOSAIKS+NL**	0.87	0.87	0.4	0.09
**Country level**	**MOSAIKS+NL**	0.79	0.79	0.29	< 0

Table S2: This is similar to the upper portion of Table S1 except that here we have evaluated on a 35 country ($\approx$ 20%) test set that was not used during model tuning. The modest reported differences in the validation-set and test-set performances when evaluating within-country performance could be due to either noise from the small sample size of the test set, or to overfitting. We test this and find that noise from the small test set is likely to be the explanation. The average within-country test-set performance across 30 random 80% validation-set and 20% test-set splits, using the same training and evaluation procedure, is very close to that of the original validation set: $R^2 = 0.51$ for MOSAIKS + NL.

		Predicted at province level (n=1,381)			
		Full variation performance		*Within-country performance*	
		ρ^2	R^2	ρ^2	R^2
HDI trained at:	***Features***	(1)	(2)	(3)	(4)
**Within-country** (n=1,363)	**MOSAIKS+NL**	0.96	0.96	0.52	0.52
	**MOSAIKS**	0.95	0.95	0.42	0.42
	**NL**	0.95	0.95	0.45	0.45
**Province level** (n=1,381)	**MOSAIKS+NL**	0.83	0.83	0.44	0.24
	**MOSAIKS**	0.76	0.75	0.0.31	< 0
	**NL**	0.60	0.60	0.44	< 0
**Country level** (n=145)	**MOSAIKS+NL**	0.74	0.74	0.28	< 0
	**MOSAIKS**	0.62	0.58	0.16	< 0
	**NL**	0.59	0.53	0.44	< 0

		Predicted at province level (n=1,381)			
		Full variation performance		*Within-country performance*	
		ρ^2	R^2	ρ^2	R^2
Life expectancy trained at:	***Features***	(1)	(2)	(3)	(4)
**Within-country** (n=1,363)	**MOSAIKS+NL**	0.92	0.92	0.05	0.05
	**MOSAIKS**	0.92	0.92	0.01	0.01
	**NL**	0.92	0.92	0.05	0.05
**Province level** (n=1,381)	**MOSAIKS+NL**	0.69	0.69	0.03	< 0
	**MOSAIKS**	0.66	0.66	0.02	< 0
	**NL**	0.43	0.43	0.06	< 0
**Country level** (n=145)	**MOSAIKS+NL**	0.6	0.57	0.02	< 0
	**MOSAIKS**	0.57	0.53	0.02	< 0
	**NL**	0.42	0.32	0.06	< 0

		Predicted at province level (n=1,381)			
		Full variation performance		*Within-country performance*	
		ρ^2	R^2	ρ^2	R^2
Mean years schooling trained at:	***Features***	(1)	(2)	(3)	(4)
**Within-country** (n=1,363)	**MOSAIKS+NL**	0.93	0.93	0.51	0.51
	**MOSAIKS**	0.91	0.91	0.41	0.41
	**NL**	0.92	0.92	0.45	0.45
**Province level** (n=1,381)	**MOSAIKS+NL**	0.75	0.75	0.49	0.43
	**MOSAIKS**	0.7	0.7	0.41	0.31
	**NL**	0.56	0.56	0.44	0.26
**Country level** (n=145)	**MOSAIKS+NL**	0.73	0.72	0.46	0.3
	**MOSAIKS**	0.6	0.6	0.24	< 0
	**NL**	0.56	0.53	0.42	< 0

		Predicted at province level (n=1,381)			
		Full variation performance		*Within-country performance*	
		ρ^2	R^2	ρ^2	R^2
Expected years schooling trained at:	***Features***	(1)	(2)	(3)	(4)
**Within-country** (n=1,363)	**MOSAIKS+NL**	0.9	0.9	0.28	0.27
	**MOSAIKS**	0.89	0.89	0.27	0.26
	**NL**	0.88	0.88	0.15	0.15
**Province level** (n=1,381)	**MOSAIKS+NL**	0.56	0.56	0.21	0.09
	**MOSAIKS**	0.5	0.5	0.2	0.06
	**NL**	0.39	0.39	0.15	< 0
**Country level** (n=145)	**MOSAIKS+NL**	0.54	0.53	0.2	< 0
	**MOSAIKS**	0.44	0.41	0.07	< 0
	**NL**	0.38	0.35	0.14	< 0

		Predicted at province level (n=1,381)			
		Full variation performance		*Within-country performance*	
		ρ^2	R^2	ρ^2	R^2
GNIpc trained at:	***Features***	(1)	(2)	(3)	(4)
**Within-country** (n=1,363)	**MOSAIKS+NL**	0.97	0.97	0.56	0.56
	**MOSAIKS**	0.95	0.95	0.4	0.4
	**NL**	0.96	0.96	0.55	0.55
**Province level** (n=1,381)	**MOSAIKS+NL**	0.79	0.79	0.36	< 0
	**MOSAIKS**	0.68	0.68	0.25	< 0
	**NL**	0.59	0.59	0.52	< 0
**Country level** (n=145)	**MOSAIKS+NL**	0.56	< 0	0.23	< 0
	**MOSAIKS**	0.46	< 0	0.1	< 0
	**NL**	0.31	< 0	0.12	< 0

Table S3: Similar to the top section of Table S1 except that here we show performance for each HDI component and also show performance with different combinations of features.

	HDI	Life expectancy	Mean years schooling	Expected years schooling
**Life expectancy**	0.79			
**Mean years schooling**	0.84	0.52		
**Expected years schooling**	0.82	0.57	0.61	
**GNIpc**	0.62	0.46	0.5	0.44
Within-country	**HDI**	**Life expectancy**	**Mean years schooling**	**Expected years schooling**
**Life expectancy**	0.31			
**Mean years schooling**	0.82	0.12		
**Expected years schooling**	0.65	0.1	0.46	
**GNIpc**	0.17	0.03	0.1	0.07

Table S4: Individual components of HDI tend to be correlated. We report the squared Pearson's correlation coefficient (ρ^2) between HDI and its components at the province level. We also report the squared correlation coefficients after demeaning provincial observations by country. This ρ^2 metric used here is intended to be comparable to the metrics reported in Tables S1 and S3. Notably, within-country correlation between HDI and GNIpc is low, yet we are still able to predict those separate outcomes with considerable skill.

		**Predicted at province level**			
		Full variation performance		*Within-country performance*	
		ρ^2	R^2	ρ^2	R^2
HDI trained at:	***Features***	(1)	(2)	(3)	(4)
**Within-country** (n=1,363)	**NDVI+NDWI+NDBI**	0.92	0.92	0.03	0.02
	**NDVI+NDWI+NDBI+MOSAIKS+NL**	0.96	0.96	0.52	0.52
	**MOSAIKS+NL** (*for reference*)	0.96	0.96	0.52	0.52

Table S5: Performance for models trained with additional features at the provincial level. Specifically, we use Sentinel 2A imagery downloaded at approximately 500m resolution [67]. Following, [18] we process Sentinel 2A imagery to calculate the Normalized Difference Vegetation Index (NDVI), Normalized Difference Water Index (NDWI), and Normalized Difference Built-up Index (NDBI). We then create population-weighted features in the same manner as done with the NL features (see Methods 5).

Supplementary figures

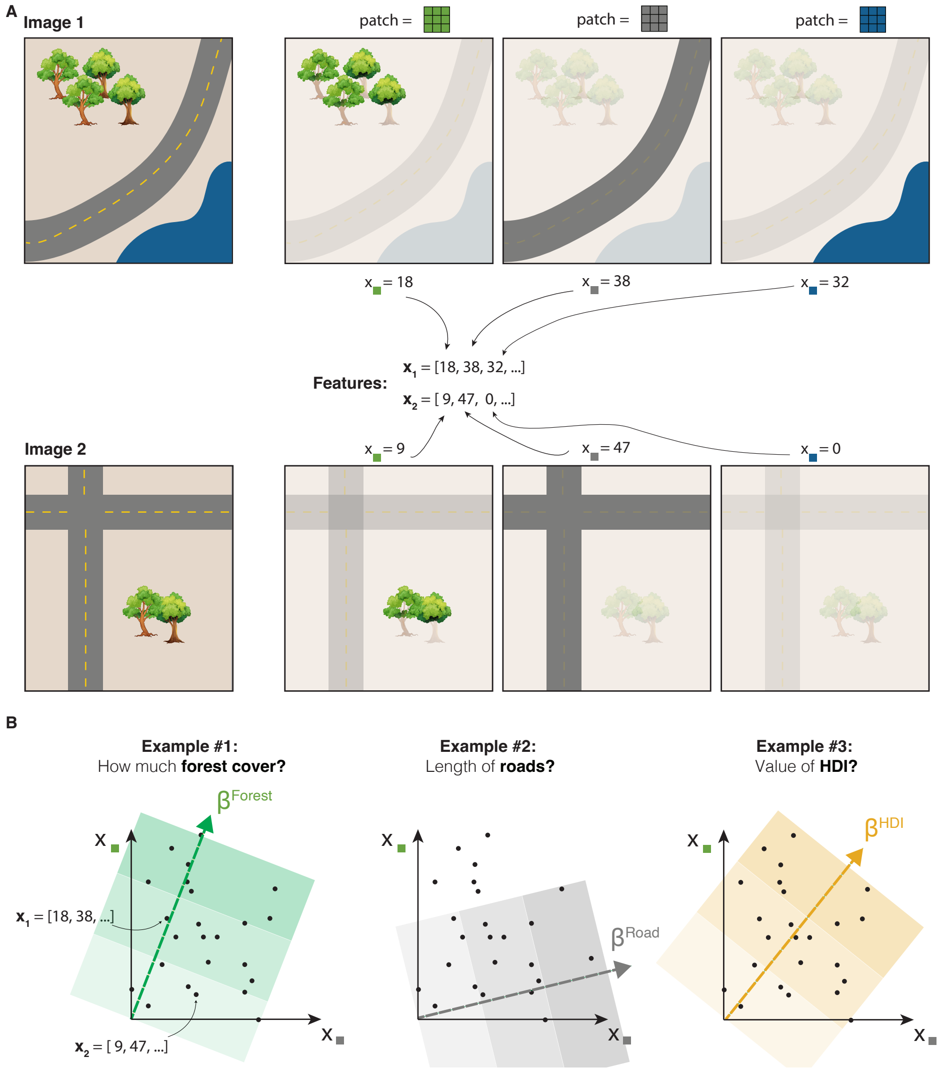

Figure S1: **An illustration of generating MOSAIKS features for two cartoon images, and using the features to predict HDI and other example outcomes.** (**A**) MOSAIKS random convolutional features capture the information within satellite imagery by measuring how similar each image is to a fixed set of small patches of imagery. Similarity is measured mathematically using a moving-window dot product, or "convolution." The parts of each image that are similar to each patch are shown, with greater similarity leading to larger feature values. The green patch, for example, is similar to the parts of the imagery containing green trees. Image 1 has a greater feature value for this patch because it has more trees than image 2. Collectively, MOSAIKS features capture information on the color and texture of the imagery, which represent the content of the imagery (e.g., trees, roads, and lakes). (**B**) Features associate differently with different outcomes: images that are more similar to the green patch tend to have higher forest cover, and images that are more similar to the grey patch tend to have more roads. Regressing HDI onto these features learns how higher or lower feature values associate with higher or lower HDI, and in turn, how to predict HDI using these features. Each dot in each scatter represents an image, and the arrow represents the direction in the feature space of increasing forest, roads, or HDI. The direction of the arrow is learned by the regression. For more details on MOSAIKS features and how they can be used to predict a broad range of outcomes see ref. [12].

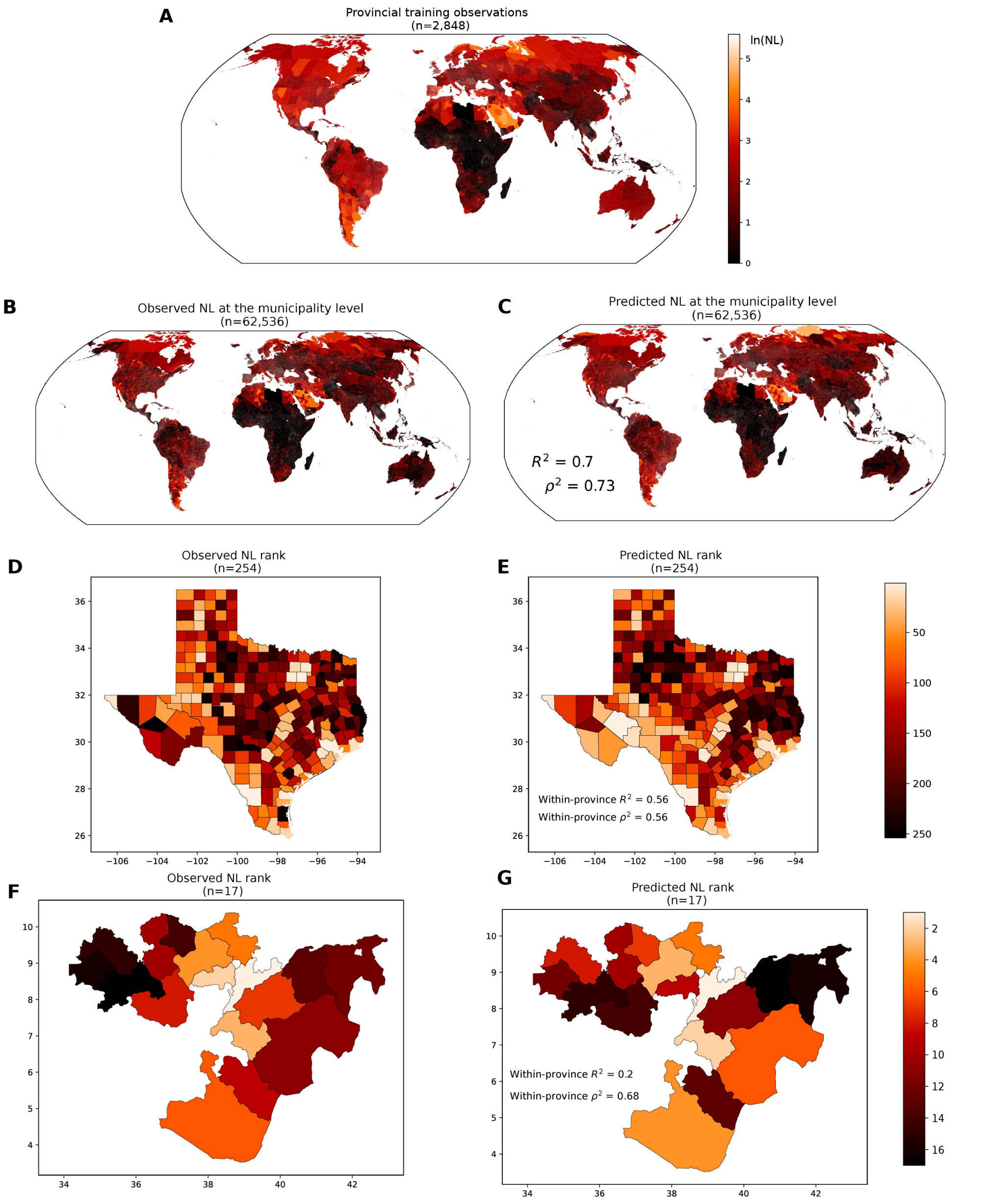

Figure S2: **A MOSAIKS model trained at the province level can effectively predict NL at the municipality level.** These maps show population-weighted NL luminosity that has been predicted using MOSAIKS. (**A**) Population-weighted NL averaged up to the provincial polygon for 2019. These are the data used to train the model. (**B**) True population-weighted NL at the municipality level. (**C**) Predicted population-weighted NL at the municipality level. (**D**) Municipalities ranked by luminosity within Texas, a single province in the United States. (**E**) Predicted nightlight luminosity rank within Texas. (**F**) Municipalities ranked by luminosity within Oromia, a single province in Ethiopia. (**G**) Predicted nightlight luminosity rank within Oromia. Panels D-G illustrate the downscaling efficacy of MOSAIKS. Each of these polygons (Texas and Oromia) represent a single training observation. All predictions come from a within-country model with predictions anchored to the country mean. Note that panels A-C use the same colorbar. See Table S1 for detailed performance metrics. The municipal shapefile used here is from geoBoundaries [65], published under a CC-BY 4.0 license.

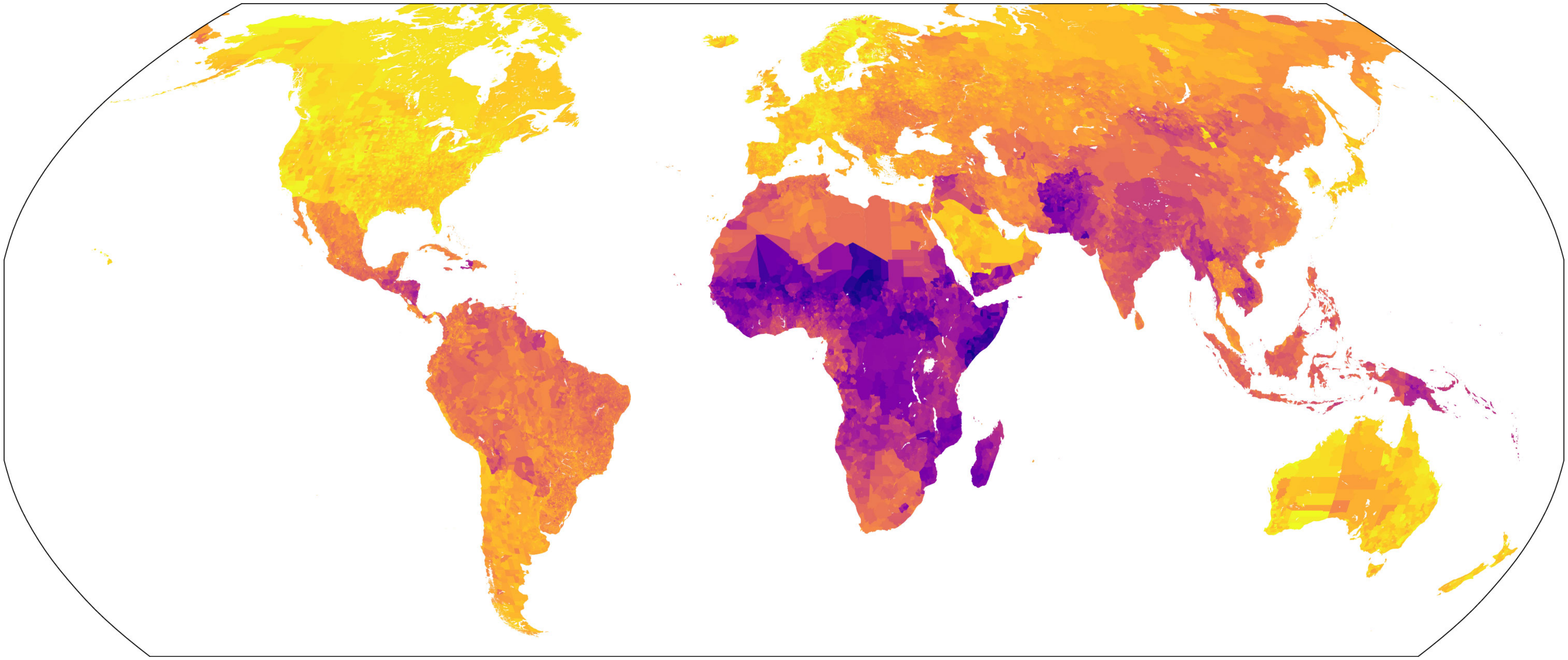

Figure S3: **Full page map of HDI estimates at the municipal level.** This is the same data as shown in Figure 3C. The municipal shapefile used here is from geoBoundaries [65], published under a CC-BY 4.0 license.

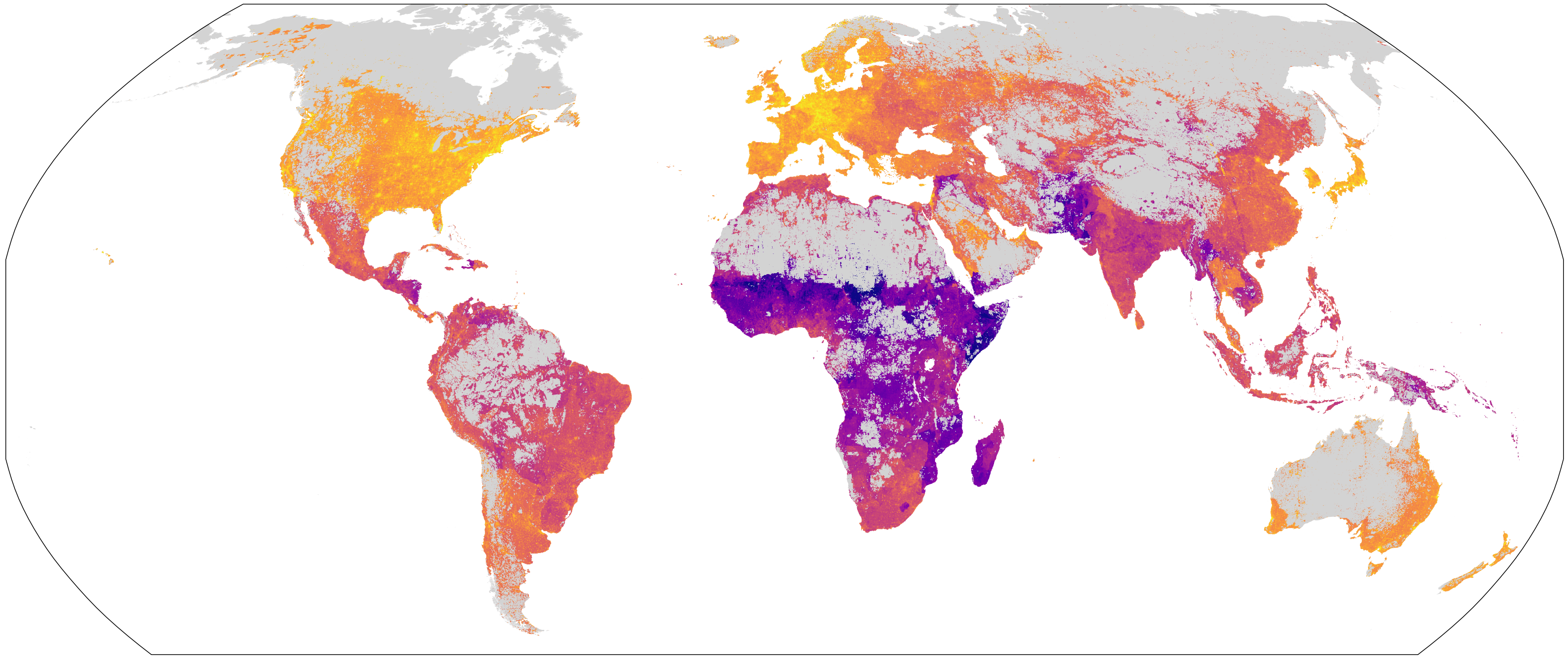

Figure S4: **Full page map of HDI estimates at the grid level.** This is the same data as shown in Figure 3D.

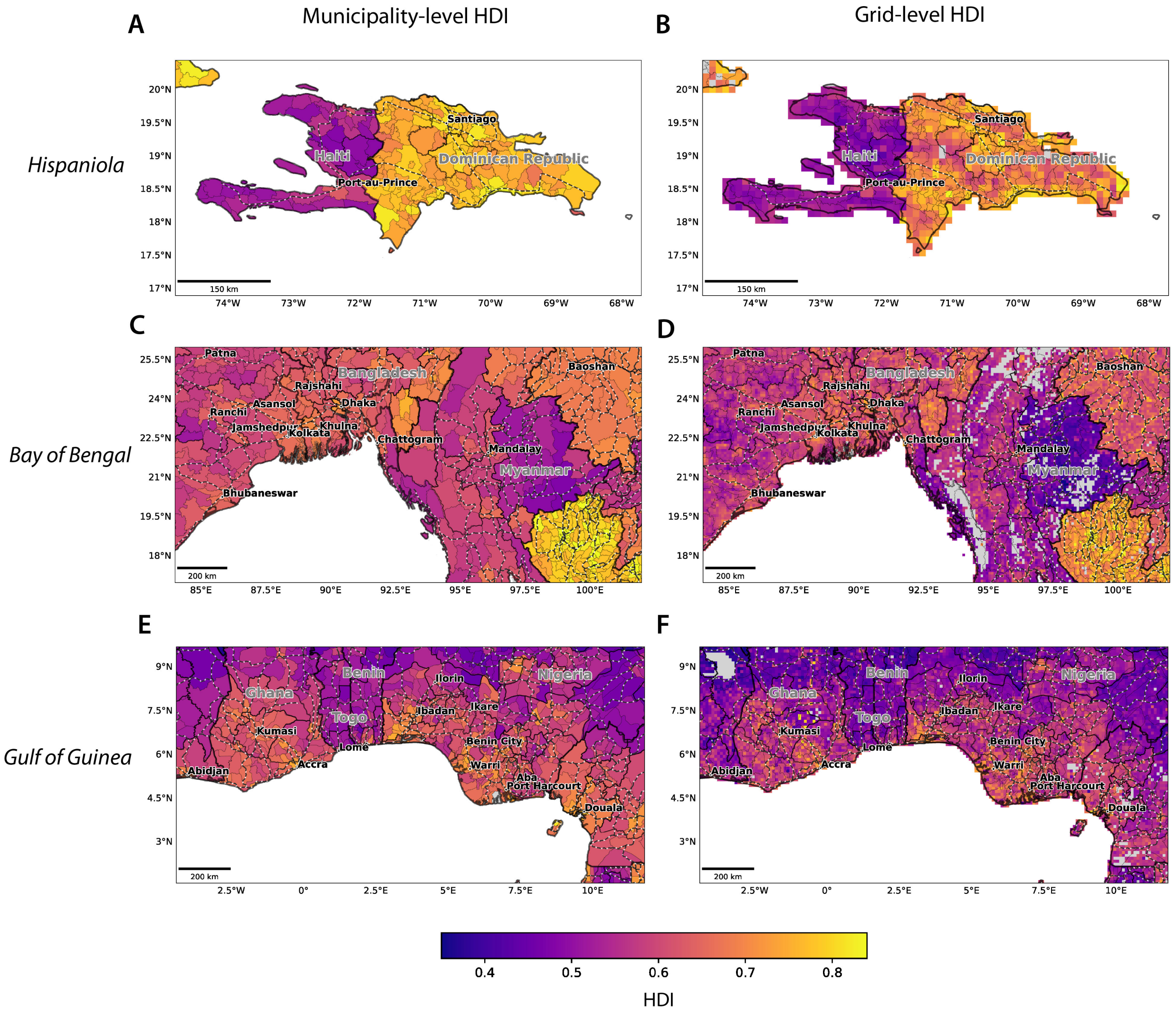

Figure S5: **Regional maps of HDI estimates at the municipal and grid levels.** (**A-B**) HDI estimates on Hispaniola (**C-D**) HDI estimates around the Bay of Bengal (**E-F**) HDI estimates around the Gulf of Guinea. All panels show country, province, and municipality borders as solid lines. Dashed lines show major roadways. Grey in the grid-level estimates indicates land area believed to be unsettled [60]. The municipal shapefile used here is from geoBoundaries [65], published under a CC-BY 4.0 license.

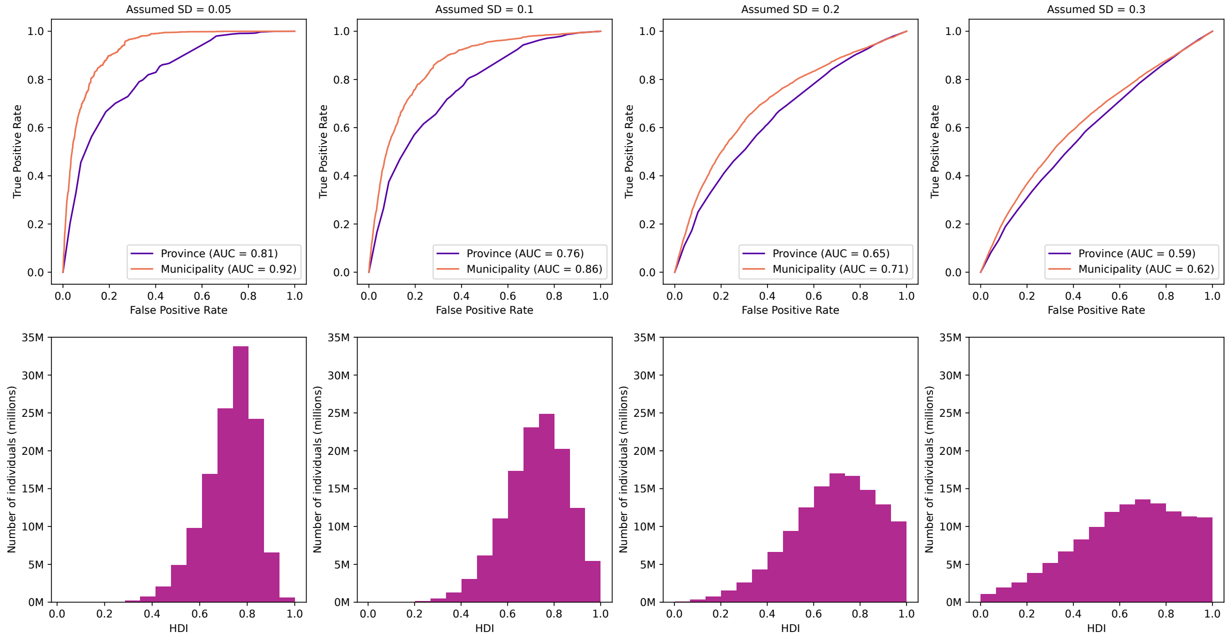

Figure S6: **The improvement in geographic targeting efficacy from using municipal values (ADM2) depends on the assumed variability of individual-level HDI within municipalities**. ROC curves as in Figure 5F for different assumed standard deviations (SD) of individual-level HDI within municipalities. Using municipal instead of provincial HDI estimates increases the AUC by 0.11 (+14% from 0.81 to 0.92) when the within-municipality HDI standard deviation is assumed to be 0.05 and by 0.06 (+9% from 0.65 to 0.71) when it is assumed to be 0.2. Histograms show the distribution of simulated individual-level HDI for each assumed SD, using a truncated normal distribution centered on the municipal values for Mexico calculated by Permanyer [9].

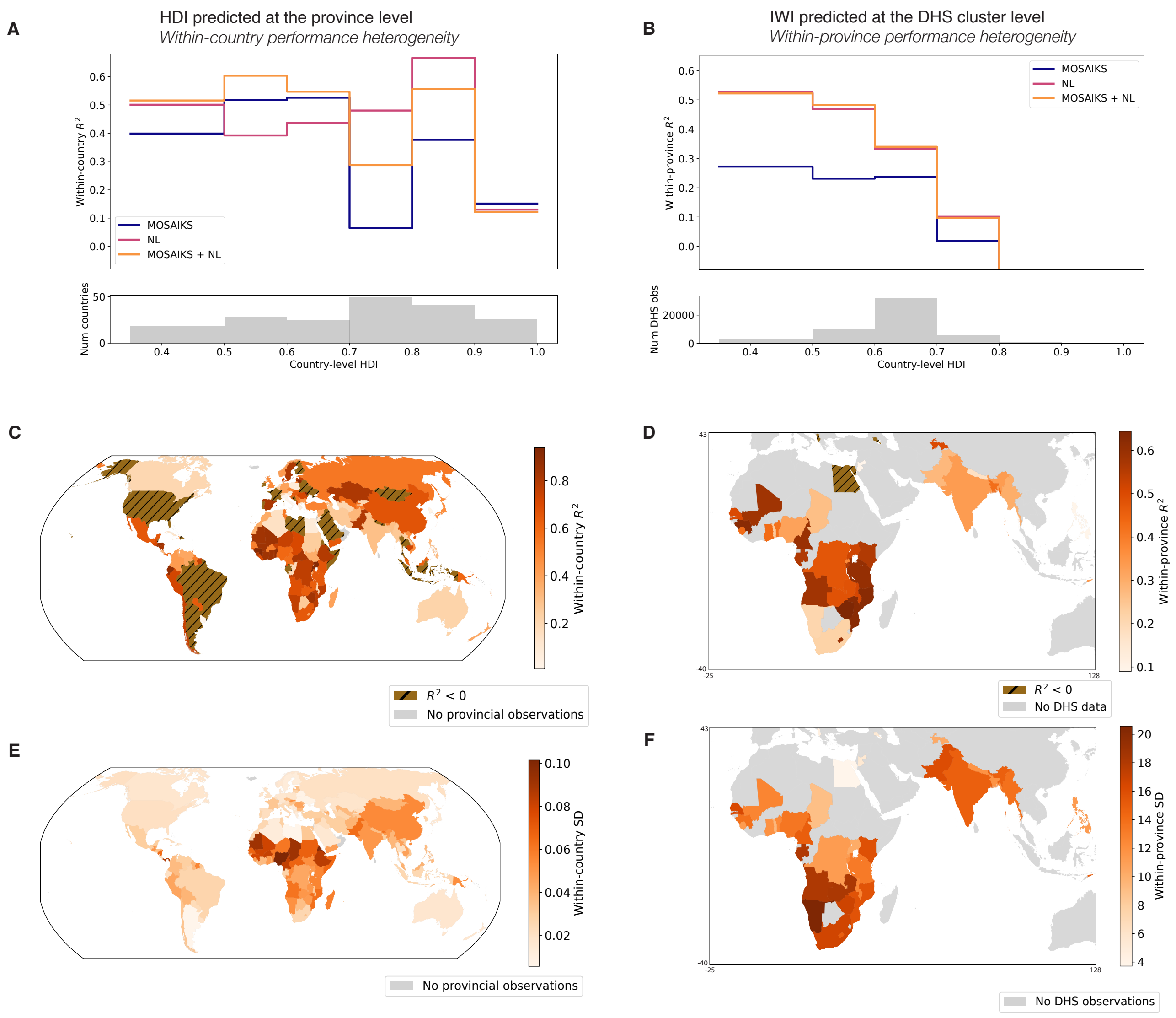

Figure S7: **Heterogeneity of HDI and IWI predictions.** On the left we show heterogeneity in HDI performance evaluated at the province level. On the right, we show heterogeneity in IWI performance evaluated at the DHS cluster level. (**A**) HDI performance as a function of parent country HDI. (**B**) IWI performance as a function of parent country HDI. (**C**) Mapped performance of HDI within-countries (within-country MOSAIKS + NL model). (**D**) Mapped performance of IWI within-provinces (within-country MOSAIKS + NL model). (**E**) Standard deviation of provincial HDI by country (**F**) Standard deviation of DHS cluster-level IWI within-provinces by country.

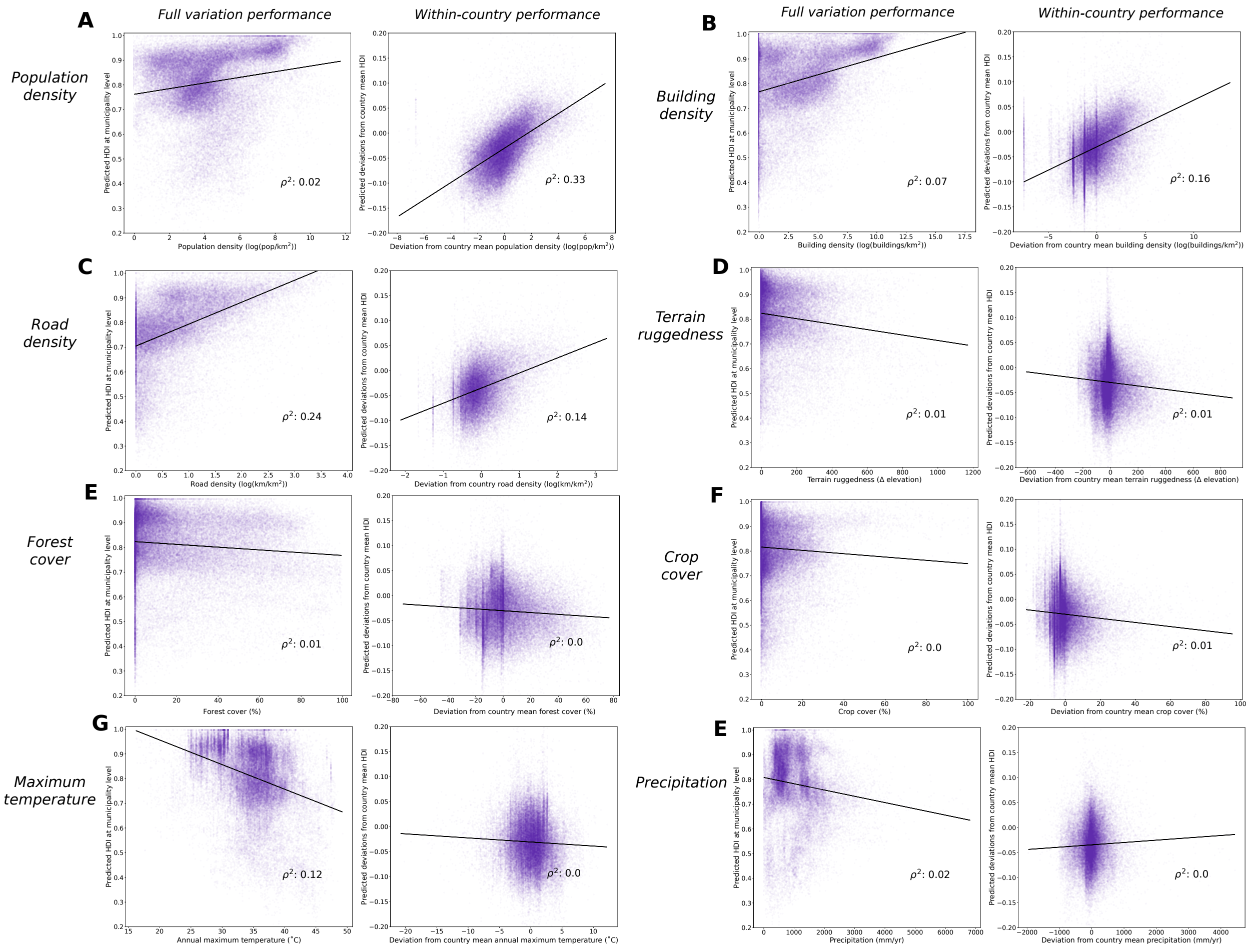

Figure S8: **Municipality-level HDI estimates can be partially predicted by other variables estimated at global scale.** Each point in each scatter represents the estimated HDI value and the value of another globally available variable for a municipality. See Supplementary Information S3 for details.

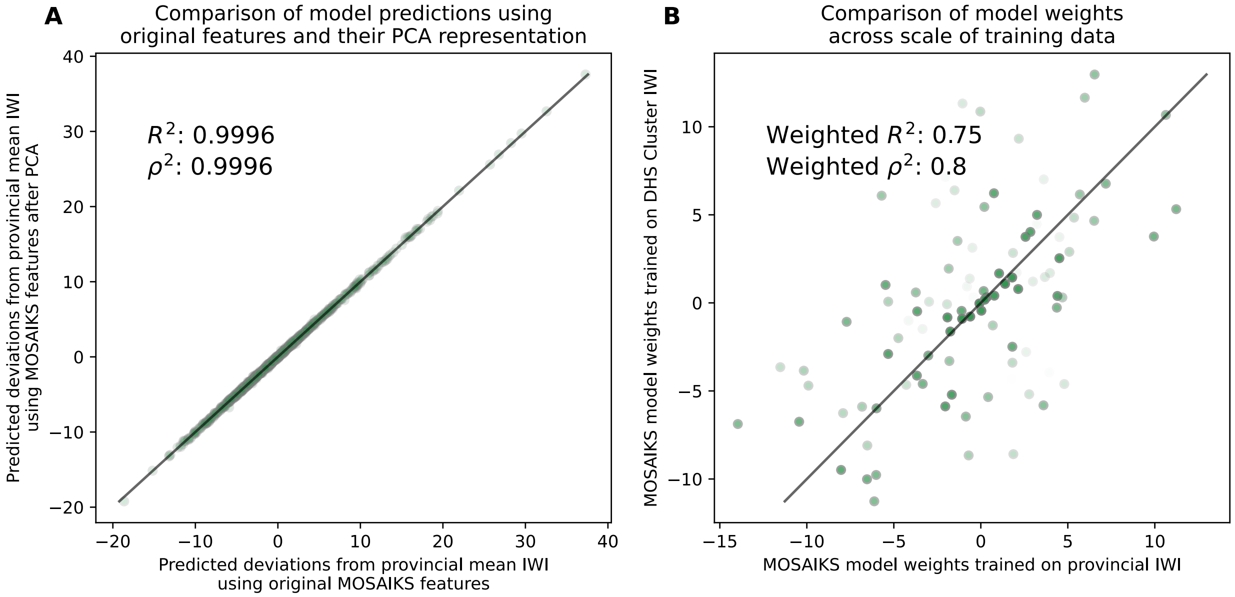

Figure S9: **Model weights for the IWI model are similar when trained at the provincial or DHS cluster level. (A)** We show that estimates of IWI are nearly identical from the model with reduced dimensionality and the primary IWI model specification. **(B)** Each point represents the contribution to the model of a single feature. The points are colored by their rank order importance after principle component analysis. R^2 and ρ^2 are weighted by the explained variance under PCA. See Supplementary Information S4 for additional details.

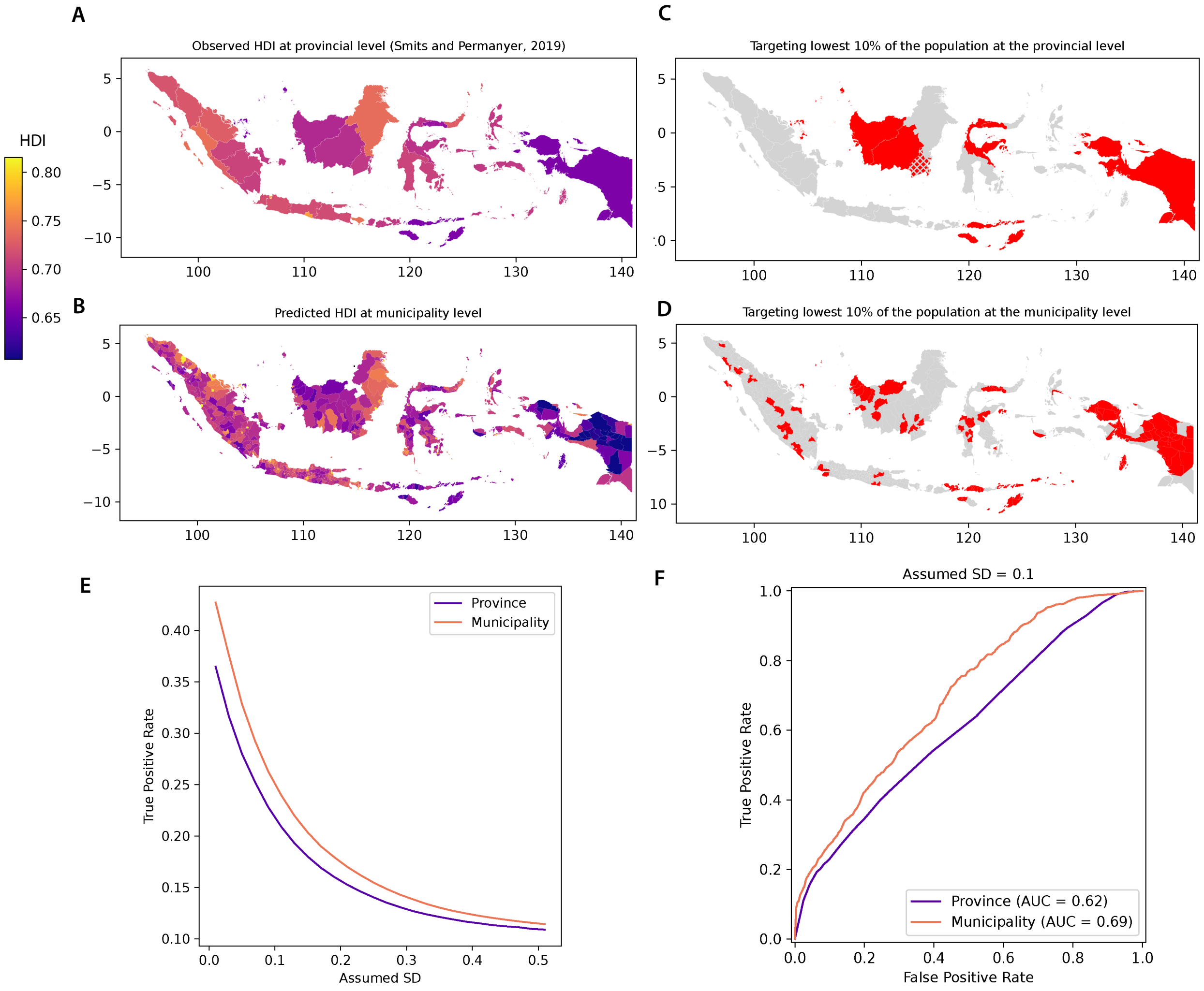

Figure S10: **Additional illustrative application: targeting policy in Indonesia.** Identical analysis as in Figure 5 using data from Indonesia. (**A**) HDI for 2019 at the province level of observation [7] (**B**) HDI estimates for 2019 at the municipality level produced in this paper. (**C**) Lowest HDI provinces that would be targeted until 10% of the country's population is reached. (**D**) Lowest HDI municipalities that would be targeted until 10% of the country's population is reached. Hashing in (**C**) and (**D**) shows the marginal province and municipality that would be partially targeted. (**E**) Targeting accuracy (true positive rate) as a function of the assumed standard deviation of HDI within each municipality. (**F**) ROC curves illustrate the degree of improvement that comes with targeting at the municipality level relative to the province level (assumed SD of 0.1 within each municipality). The municipal shapefile used here is from geoBoundaries [65], published under a CC-BY 4.0 license.

S1 Supplementary discussion of the co-production of the analysis and estimates of HDI

This analysis and the associated estimates of the UN HDI were co-produced by a team of academics and practitioners aiming to develop measures of HDI that reflect the understanding and needs of both practitioners and policymakers [68, 69]. The effort was initiated by researchers and practitioners from the United Nations Development Programme (UNDP), coordinated by Dr. Heriberto Tapia, head of research for the Human Development Report Office and a co-author of this study. The Human Development Report Office is responsible for producing and publishing official HDI statistics for the United Nations. Members of the UNDP approached academic members of the team in 2021, with the goal of increasing the resolution of the HDI globally based on the growing demand for consistent disaggregated data following an impartial global standard. The combined team (practitioners and academics) jointly refined a project design and secured grants from third-parties and funding from the UNDP. Project implementation, writing, and dissemination was a joint and iterative process involving all team members.

Throughout the project, the team worked deliberately to ensure mutual knowledge transfer, with the academics learning what applications and aspects of modeling are most important to practitioners, and the practitioners learning the technical aspects of the model's development. The practitioners and academics collectively identified model performance as a primary priority, with model generalizability, simplicity, transparency and interpretability as additional design goals. Motivated by the results of this project, the UNDP is engaging in follow-on work that predicts the United Nations Multidimensional Poverty Index using the methods developed here. Academic members of our team are supporting and advising this follow-on work in an effort designed to solidify the transfer of this technology to UNDP and to build UNDP's technical capacity internally.

The UNDP team is particularly interested in satellite-based measurements of HDI for three reasons. First, satellite-based measurements adhere to relatively impartial technologically-led standards that facilitate international comparisons, a key attribute of UN indicators. Second, the integration of satellite imagery with machine learning creates new opportunities for using the HDI in policymaking. In the short term, this new data can facilitate planning and resource allocation within countries. In the medium term, these estimates should become a key input for analyzing the interaction between socioeconomic and geophysical data in studying the effects of climate change on people. This will be crucial for designing a new generation of policy responses through a global lens. Third, the availability of a database and a replicable method for generating these estimates is expected to address equity issues.

In a rapidly changing world, there is an increasing need for information for decision-making. Communities with high levels of human development tend to have high-quality surveys and the resources to power a data revolution. In contrast, communities with low human development, where additional data is most needed, tend to face substantial constraints in both surveys and analytical capacity. The availability of both a database and a standard, replicable method for generating these estimates is expected to be a valuable global public good.

S2 Supplementary discussion of model performance

Model performance across regions Analyzing the performance of MOSAIKS models across space, we find that performance tends to be the highest in low income regions, especially sub-Saharan Africa, where such measurements are likely to be of greatest value (Figure S7A,C). Specifically, we find that MOSAIKS models explain more variation of province-level HDI deviations from the country mean in areas of low human development (HDI < 0.6, $R^2 = 0.57$) than in areas of medium or high human development (HDI > 0.6, $R^2 = 0.43$, Figure S7A). We also see this pattern in predictions of DHS cluster-level IWI deviations from the province mean, with performance increasing monotonically from $R^2 = 0.09$ for countries with the highest HDI values to $R^2 = 0.52$ for countries with the lowest HDI values (Figure S7B,D). This improved performance may be due to increased variance of HDI and IWI values within these countries (Figure S7E-F), which provides more variation to exploit during model training. Alternatively, variations in wellbeing being may be relatively easier to see from satellite imagery in areas with lower human development. Relatedly, model performance for both HDI and IWI is higher in regions with higher inequality, highlighting the particular value of these estimates in regions of high inequality ($p < 0.01$ for both HDI and IWI; pearson's ρ is 0.33 for HDI and 0.53 for IWI comparing the within-country standard deviation of provincial values and the within-country predictive performance, measured by ρ^2).

Motivated by these differences in performance across regions, we test whether the relationship between image features and HDI varies spatially. We do so by training an additional model that allows the MOSAIKS features to have a continent-specific relationship with provincial HDI. We observe a small drop in within-country performance relative to the baseline model (from $R^2 = 0.52$ to 0.49), indicating that increasing the complexity of the model in this way does not improve out-of-sample performance.

Value from combining daytime and nighttime imagery The MOSAIKS-based approach can use image features from multiple sensors simultaneously when training models, a property that is used throughout this analysis to predict HDI from both daytime and nighttime imagery. Analyzing the performance of MOSAIKS models based on the type of satellite imagery used, we find that daytime and nighttime imagery together explain 7% more variation in provincial HDI deviations from the country mean than does nighttime imagery alone, improving model fit by 16% from $R^2 = 0.45$ to 0.52 (Table S3). This improved performance from using daytime and nighttime imagery together is strongest in regions of low human development (HDI < 0.6) (Figure S7A), consistent with a previous finding that models using daytime imagery outperform models using nighttime imagery when predicting the consumption and assets of populations with the lowest consumption and assets in five African countries [16]. Analyzing model performance for each component of HDI, we see that the improved performance predicting HDI using daytime and nighttime imagery stems from improved or comparable performance predicting each component of HDI (largest change in R^2 is 0.12 for expected years of schooling, smallest is no change in R^2 for life expectancy, Table S3).

In exploratory analysis of subsamples, we have observed that in several middle income countries with HDI between 0.7 and 0.8 (including those where we have municipal variation in HDI), within-country R-squared is sometimes higher when models are trained on nighttime imagery alone, relative to models that combine MOSAIKS and nighttime imagery. However, for other parts of the HDI distribution and on average, as described above, MOSAIKS features appear to add performance to the full model. We currently cannot explain why a nightlights-only model performs best for these particular countries when predicting HDI, and we do not know if this phenomena extends to non-HDI outcomes. In future work, we hope to present a more complete analysis that more fully evaluates how data from many satellite sensors, including others not used in this analysis, can be effectively and efficiently combined in SIML applications.

Model performance training at the country-level To evaluate performance in an extremely data-limited setting, we re-train our model using only country-level data. Despite a low number of training observations (N = 85 to 170 across experiments) these models maintain 36% to 54% of the performance of our preferred models trained using provincial deviations from the country mean (N = 862 to 2,852) when evaluated on the relative ordering of predicted and observed values using ρ^2 in all experiments (Table S1). This indicates that our approach can achieve competitive predictive performance detecting locations with relatively higher and lower HDI even when trained on few and coarse observations of the

variable of interest. Performance predicting the exact level of HDI is lower, especially when evaluated within-country, likely due to the large difference in the magnitude of HDI variation across countries versus within countries, discussed above (Figure 2A,B).

S3 Supplementary discussion of model transparency and interpretability

The models employed in this analysis were designed to maximize predictive performance, based on co-development with our team members at the United Nations Development Programme (Supplementary Information S1) and feedback from other practitioners, who generally identified model performance as the most important property of the model. Other important aspects of model development are model transparency and interpretability [55] (Figures S1 and S8).

Model transparency Model transparency is the ability to clearly convey the model, its design, and its estimation [55]. Transparency is a strength of the MOSAIKS approach used and developed here. The features are can be precisely described mathematically [12] and conveyed simply: each feature measures the similarity between a small patch of imagery and the image of interest, where similarity is measured using a moving dot product (i.e., convolution) of the patch over the imagery. This is illustrated in the cartoon developed in Figure S1, which shows how three example patches capture the amount of trees, roads, and lakes in imagery. One explanation of how the MOSAIKS approach works is that the model predicts HDI in new areas where HDI is not known by assigning the (weighted) average value of HDI from places where HDI is known that look similar in the imagery (see Supplementary Note 2.3 in [12] for details). The linear model structure is also straightforward, especially compared with more complicated structures like a deep convolutional neural network or gradient boosted decision tree. We relate the image features to HDI using a linear ridge regression, which, unlike many more complex models, has an analytical solution. The simplicity and transparency of the MOSAIKS approach makes it straightforward to fully describe, understand and use [12]. The work in this manuscript extends the ability of the MOSAIKS approach to learn the relationship between imagery and an outcome of interest using labels from any set of political boundaries, while maintaining a transparent design.

Model interpretability Model interpretability is the ability to understand what aspects of the input data are responsible for model predictions [55]. To better interpret what aspects of the imagery are captured by our HDI estimates, we examine whether our municipality

HDI estimates are correlated with known variables that can be constructed at the municipality level (Figure S8). Specifically, we construct municipality-level estimates for population density, road density, building density, terrain ruggedness, forest cover, crop cover, maximum temperature, and precipitation. Data for each of these variables is publicly available. We create a measure of each variable at the municipal level using the source data product and our municipality (ADM2) shapefile from geoBoundaries [65].

Population density Municipal population density data were constructed as the population count from GHS-POP divided by the area of the municipality.

Building density Building density data at the municipality level were constructed using the gridded building data product from Microsoft Bing Maps ($\approx 0.2 \times 0.2$ degree resolution) [70]. This data is relatively coarse compared to the municipality size, so we assume a uniform distribution of buildings within the source raster when averaging building density over municipal polygons. We take this approach for all raster datasets used in this interpretability analysis.

Road density Municipal road density estimates were constructed by calculating the length of road in each municipal polygon and dividing by the polygon area. Road data are from Open Street Maps [71]. To facilitate computation we calculate road density over a 1% sample of global land area before averaging to the municipal level ($\approx$1 km^2 sampled every $\approx$10km in the North-South and East-West directions). Municipality observations that do not contain a sampled road density observation are dropped from this analysis.

Terrain ruggedness Municipal ruggedness data were constructed using the global raster created by Shaver et al. [72] at 1km $\times$ 1km resolution. In this dataset "the ruggedness of any given 1 km^2 area is determined by measuring how the average elevation of that area differs from all those of neighboring 1 km^2 areas" [72]. Municipal values are calculated as the average of ruggedness values within each municipality.

Forest cover Forest cover data come from the Hansen et al. data product [73], which is available at 30m $\times$ 30m resolution. Municipal estimates are calculated as the average forest cover over the municipal polygon.

Crop cover Crop cover data come from the Ramankutty et al. data product available at 0.08×0.08 degree resolution [74, 75]. For each municipality we calculate the fraction of the polygon area covered by cropland.

Maximum temperature We construct a measure of typical maximum annual temperature from the Climate Prediction Center gridded data product at 0.5×0.5 degree resolution [76]. Specifically, we retrieve roughly a decade of daily data (2007-2018) and calculate the maximum temperature observed in each grid cell each year. We then take the average across years and over each municipal polygon.

Precipitation Precipitation data are from the NASA IMERG annual average precipitation data product at 0.1×0.1 degree resolution [77]. We calculate municipal values as the average of gridded values over each municipal polygon.

To estimate the fraction of variance explained by each of these variables individually we execute a simple linear regression of our municipal HDI estimates on each variable. Within-country estimates are calculated after demeaning the HDI estimates and each variable by country. To estimate the fraction of variance explained by all of these variables together we execute a multiple linear regression of our municipal HDI estimates on all variables together. Before estimating this latter regression we used mean-imputation to fill in any missing values.

S4 Supplementary discussion assessing consistency of model weights across spatial scales

A key feature of our approach is that it can be trained on and make predictions for units of arbitrary shape and size. We employ this approach to train on global provincial HDI data and make predictions of HDI for global municipalities and a $0.1^{\circ} \times 0.1^{\circ}$ grid. For this approach to be effective, model weights estimated at the provincial level, must be able to make skillful predictions at the municipal and grid levels.

One way to understand how this approach works is to see that, in a linear model, the relationship between aggregated outcomes and aggregated features should be similar to the relationship between disaggregated outcomes and features. This is illustrated in Equations 2-3 of the main text. Rolf et al [12] provide additional mathematical explanation for relating predictions made using MOSAIKS features at image and sub-image scales. A primary goal of this manuscript is to propose that this approach can be used to address the challenge of limited training data in remote sensing applications by allowing for training on irregularly structured and sized observations – which is not discussed in ref. [12] – and to empirically test whether this works in practice. The primary evidence supporting this are the downscaling tests reported in Figure 2 and Table S1.

Here, we additionally explore the question of *why* the approach works by empirically testing whether model weights estimated at aggregated and disaggregated scales are similar. To do so, we compare model weights between models of IWI trained at the provincial (N = 862) and DHS cluster (N = 51,996) scales. We use IWI for this experiment because there are a large number of aggregated and unaggregated observations that span the same spatial extent.

A challenge in designing this experiment is that MOSAIKS features are correlated with each other, and there are a large number of features relative to the number of training observations. This means that the same information could load onto different features even when training and retraining at the same scale if we do not introduce additional constraints to the feature set. Put another way, different sets of model weights could give the same predictions and represent the same relationship between the imagery and outcome of interest. This is not an issue in our main application, since the set of weights obtained at an aggregated scale will remain valid if applied to a disaggregated scale, and vice versa. However, there is no guarantee that the same weights will be obtained if models are independently fit at both scales, since there are multiple valid ways to represent the data using the model features. Thus, for this experiment, we first transform our features into an orthogonal basis.

We use Principal Components Analysis (PCA) to project the MOSAIKS features into a feature space with independent (i.e., uncorrelated) features that contain the same information as the original features, following ref. [13]. PCA can also be used to reduce the dimensionality of the feature space, which can aid interpretation in this setting by focusing on features that explain most of the variation in the imagery, and thus likely in the outcome of interest. We find that 100 PCA features explain $> 99.9\%$ of the variation in the original 4,000 MOSAIKS features in this context, and that a model trained using these 100 PCA features provides essentially identical predictions to a model trained using the 4000 original features (Figure S9A). This PCA model is thus practically identical to our MOSAIKS-based model but with independent features that represent an orthogonal basis. We use these orthogonalized and rotated MOSAIKS features to analyze whether model weights are consistent when training at different levels of aggregation.

Using these orthogonal MOSAIKS features, we find that model weights estimated using the (aggregated) provincial data are very similar to model weights that are independently estimated using (disaggregated) DHS cluster data (R^2=0.75, Figure S9A). Note that when calculating R^2 in this setting we weight by the fraction of variance in the MOSAIKS features each component explains, so that greater weight is placed on features that explain more variation in the MOSAIKS features, and likewise, in IWI. The high correspondence between weights estimated at aggregated and disaggregated scales indicates that the same satellite

information is being used in the same way to predict IWI at both scales. This helps to explain how our approach is able to achieve skill in the downscaling applications illustrated in Figure 2 and Table S1.

S5 Supplementary discussion on using alternative image indices

To evaluate whether additional satellite data sources beyond visual daytime imagery and nighttime imagery might improve HDI estimates, we follow ref. [18] and add to the baseline model three additional index-based features constructed from Sentinel-2A satellite imagery that integrate knowledge of spectral properties of different ground conditions: the Normalized Difference Vegetation Index, Normalized Difference Water Index, and Normalized Difference Built-Up Index. We find that the model performance is essentially unchanged (Table S5), indicating that these additional features do not provide additional information beyond what is already captured by the MOSAIKS and NL features. While it is important to note that there is a limit to how well socioeconomic variables can be predicted using satellite imagery generally, and adding these additional features did not improve performance in this case, future work should nonetheless explore whether incorporating additional imagery sources and/or other ancillary data can improve these estimates [78].

S6 Supplementary methods

Note that in the supplementary methods we use the subscript p to refer to provincial or first-level administrative regions; and the subscript m to refer to municipality or second-level administrative regions. We use the subscript c to denote observations at the country level.

S6.1 HDI model training

Within-country model training Because our focus is explaining subnational variation in HDI, we specifically train our primary model to predict within-country deviations of HDI. To do this, we first demean subnational observations by country and then train a model to use imagery to predict these residualized deviations. Specifically, we transform observed ADM1 HDI for province p (HDI_p^{ADM1}) into the deviation of this value from the country mean HDI ($\widetilde{HDI}_p^{ADM1}$). We then solve a ridge regression to predict $\widetilde{HDI}_p^{ADM1}$ based only on

provincial daytime ($\widetilde{\mathrm{X}}^{ADM1}_{MOSAIKS,p}$) and nightlight ($\widetilde{\mathrm{X}}^{ADM1}_{NL,p}$) features that have been similarly residualized relative to the country mean values for these variables. We learn the model

$$\widetilde{HDI}_p^{ADM1} = \beta_0 + \boldsymbol{\beta}_1 \, \widetilde{\mathrm{X}}^{ADM1}_{MOSAIKS,p} + \boldsymbol{\beta}_2 \, \widetilde{\mathrm{X}}^{ADM1}_{NL,p} + \epsilon_p \tag{S1a}$$

where :

$$\widetilde{HDI}_p^{ADM1} = HDI_p^{ADM1} - \sum_{p \in c} \frac{HDI_p^{ADM1}}{N_c} \tag{S1b}$$

$$\widetilde{\mathrm{X}}^{ADM1}_{MOSAIKS,p} = \mathrm{X}^{ADM1}_{MOSAIKS,p} - \sum_{p \in c} \frac{\mathrm{X}^{ADM1}_{MOSAIKS,p}}{N_c} \tag{S1c}$$

$$\widetilde{\mathrm{X}}^{ADM1}_{NL,p} = \mathrm{X}^{ADM1}_{NL,p} - \sum_{p \in c} \frac{\mathrm{X}^{ADM1}_{NL,p}}{N_c}. \tag{S1d}$$

Here, N_c is the number of provinces in country c. Note that we restrict predictions from this demeaned model to be between the observed minimum and maximum HDI deviations from the country mean.

Anchoring to country means via re-centering To evaluate full variation performance using the within-country model (Table S1, col. 1-2) we need HDI predictions in "levels" rather than predicted deviations from the country mean. To construct predicted HDI values in "levels" we anchor our estimates to country means, since they are observed and used in the estimation procedure. Practically, this means we add the country mean HDI, which was subtracted from the observations before model training, back onto the predicted deviations:

$$\widehat{HDI}_p^{ADM1} = \widehat{\widetilde{HDI}}_p^{ADM1} + \sum_{p \in c} \frac{HDI_p^{ADM1}}{N_c} \tag{S2}$$

Note that it is not necessary to implement this procedure when evaluating within-country performance.

Province and country model training In Table S1, we additionally report performance for models trained on province and country-level data directly. Unlike the within-country

model, these models are trained on values in "levels" instead of deviations from the country mean. In these experiments, we learn the models:

Province model:

$$HDI_p^{ADM1} = \beta_0 + \beta_1 X_{MOSAIKS,p}^{ADM1} + \beta_2 X_{NL,p}^{ADM1} + \epsilon_p \tag{S3}$$

Country model:

$$HDI_c^{ADM0} = \beta_0 + \beta_1 \ X_{MOSAIKS,c}^{ADM0} + \beta_2 \ X_{NL,c}^{ADM0} + \epsilon_c \tag{S4}$$

We do not apply a mean-anchoring procedure with these models as their predictions are already in "levels" rather than predicted deviations. Note that 20 of the 179 total countries do not have subnational data (e.g., Qatar) and that these 20 country-only observations are included in both province and country models.

S6.2 Downscaling validation with IWI

Labels IWI is similar to the wealth index reported in DHS surveys, except that it was created to be comparable across countries [37]. IWI data are available both for provincial polygons, which we use for training, and for DHS clusters, which we use for evaluation. For each survey cluster, DHS provides coordinate points associated with the cluster centroid. To protect privacy, the actual GPS coordinates of the center of each cluster are randomly displaced by up to 2km for urban clusters and up to 5km for rural clusters, with a random 1% of rural cluster coordinates displaced by up to 10km. According to DHS, the displaced coordinate is guaranteed to fall within the same DHS-provided administrative boundaries as the true cluster centroid. To map these point observations to administrative polygons, we spatially buffer urban cluster coordinates using a 2km radius and rural cluster coordinates using a 10km radius. We then clip these buffers to the finest DHS-provided administrative boundaries that are available.

Training We train within-country, province level, and country level IWI models following the structure of models for HDI (Methods Section S6.1). Provincial IWI observations are denoted IWI_p^{ADM1}.

The within-country IWI model, our preferred model specification, takes the same form as Equation S1a:

$$\widetilde{IWI}_p^{ADM1} = \beta_0 + \beta_1 \ \widetilde{X}_{MOSAIKS,p}^{ADM1} + \beta_2 \ \widetilde{X}_{NL,p}^{ADM1} + \epsilon_p \tag{S5a}$$

where :

$$\widetilde{IWI}_p^{ADM1} = IWI_p^{ADM1} - \sum_{p \in c} \frac{IWI_p^{ADM1}}{N_c} \tag{S5b}$$

Note that $\widetilde{X}^{ADM1}_{MOSAIKS,i}$ and $\widetilde{X}^{ADM1}_{NL,i}$ are the same feature matrices defined in Equation S1c and S1d but with a different number of observations due to differing availability of outcome data.

Prediction We evaluate the IWI model performance at a finer resolution than it was trained. We use the trained provincial model (Equation S5a) to produce predictions of IWI at the DHS cluster level and compare those predictions to the cluster-level IWI measurements from the GDL, which were not used for model training. We calculate DHS cluster-level features in the same way as for the other administrative polygons.

To make predictions of IWI deviations from the country mean at the DHS cluster level using the within-country model trained on provincial deviations from the country mean, we multiply model weights with the demeaned DHS cluster-level satellite features:

$$\widehat{\widetilde{IWI}}_d^{DHS} = \hat{\beta}_0 + \hat{\boldsymbol{\beta}}_1 \, \widetilde{X}^{DHS}_{MOSAIKS,d} + \hat{\boldsymbol{\beta}}_2 \, \widetilde{X}^{DHS}_{NL,d} \tag{S6}$$

where d indexes DHS cluster and $\hat{\beta}_0$, $\hat{\boldsymbol{\beta}}_1$, and $\hat{\boldsymbol{\beta}}_2$ are estimated in Equation S5a. Tildes denote that these predictions are predicted deviations from the country mean. In our within-country IWI model, we demean DHS cluster-level satellite image features by the same country average feature values as in the training procedure:

$$\widetilde{X}^{DHS}_{MOSAIKS,d} = X^{DHS}_{MOSAIKS,d} - \sum_{p \in c} \frac{X^{ADM1}_{MOSAIKS,p}}{N_c} \tag{S7a}$$

$$\widetilde{X}^{DHS}_{NL,d} = X^{DHS}_{NL,d} - \sum_{p \in c} \frac{X^{ADM1}_{NL,p}}{N_c} \tag{S7b}$$

where we note that these averages are constructed by averaging province-level features, but have similar values that averages of nationally-representative sets of cluster-level features would have.

Anchoring to provincial means via re-centering To construct estimates of cluster-level IWI in levels ($\widehat{IWI}_d^{DHS}$), we anchor predicted cluster-level deviations from the country mean ($\widehat{\widetilde{IWI}}_d^{DHS}$) to the known provincial value (IWI_p^{ADM1}) using a provincial level adjustment:

$$\widehat{IWI}_d^{DHS} = \widehat{\widetilde{IWI}}_d^{DHS} + \underbrace{IWI_p^{ADM1} - \sum_{d \in p} \frac{\widehat{\widetilde{IWI}}_d^{DHS}}{N_p}}_{\text{centers DHS clusters to known provincial values}} \tag{S8}$$

Here, N_p denotes the number of DHS clusters contained by ADM1 polygon p, and IWI_p^{ADM1} denotes the observed ADM1-level value for polygon p. This anchors the mean of our DHS cluster-level predictions within each provincial polygon to the respective known province value used in training.

S6.3 Downscaling validation using nighttime lights as labels

In our analysis of the downscaling perfomance of our approach, we design an experiment in which NL are used as *labels* and are *not used as features* (Figure S2). This experiment is useful because it is the only validation experiment where the ground truth data are available globally and at municipal resolution. Thus, this experiment allows us to evaluate predictions at a downscaled resolution for the entire globe using a procedure that mirrors how we will generate downscaled HDI estimates (such global high resolution labels do not exist for our other outcomes). We do not expect NL predictions to be perfect proxies for HDI data in this regard, but if NL can be downscaled successfully, it provides support for the *procedure* we use to downscale HDI.

Labels We use population estimates from GHS-POP and fine resolution NL data from VIIRS to create a population-weighted average NL radiance at the province level. NL observations are population-weighted to mirror the construction of HDI, which is also population-weighted. Combining NL observations with population is also common practice when using NL as a development indicator [42, 44, 45]. We construct municipality-level NL observations using a municipal (ADM2) shapefile from geoBoundaries [65], which links municipalities to provincial "parent" polygons.

We exclude Ireland from the geoBoundaries ADM2 dataset because Irish municipalities (ADM2 units) are so small that they alone represent 45% of the global municipality observations. Thus, they would be over-represented in global performance metrics relative to their

size if not removed.

The vertical streaking patterns in the scatter plots in Figure 2H-J are caused by other countries that also have very spatially dense municipalities, though not to the same degree as Ireland. Because many within-province predictions are clipped at the observed minimum or maximum within-country deviation, this creates vertical streaking at the extremes in 2J. When the country-level mean values are added back, this results in vertical streaking at an arbitrary point along the x-axis in 2H-I. The three countries that mostly account for this effect are Great Britain (≈ 9,000 units), Spain (≈ 8,000 units), and Brazil (≈ 5,000 units).

Training We train a model using only MOSAIKS features constructed from daytime imagery to predict NL:

$$NL = \beta_0 + \boldsymbol{\beta}_1 \, \mathbf{X}_{MOSAIKS} + \epsilon \tag{S9}$$

This model structure is broadly the same training procedure described in Methods Section S6.1 and in Equation 4; however, we do not include NL features when predicting average NL luminosity. NL is also now a vector of scalar NL observations rather than a matrix of features.

Prediction To generate municipal predictions, indexed by m, from the within-country model, we first create municipal predictions of NL deviations from the country mean. We demean $\mathrm{X}^{ADM2}_{MOSAIKS,m}$ by country by subtracting the country mean feature values and then multiplying the resulting demeaned features by the estimated model weights. This corresponds to what is done when evaluating downscaled IWI performance in Section S6.2 and shown in Equation S7a.

Anchoring to country means via re-centering When converting the predicted municipal NL deviations from the country mean ($\widehat{\widetilde{NL}_m^{ADM2}}$) into predicted municipal NL values in levels ($\widehat{NL_m^{ADM2}}$), we anchor values to the known country mean:

$$\widehat{NL_m^{ADM2}} = \widehat{\widetilde{NL}_m^{ADM2}} + \sum_{p \in c} \frac{NL_p^{ADM1}}{N_c} \tag{S10}$$

Note that we anchor fine resolution NL predictions to the known country mean rather than the provincial mean (following Equation S2 rather than Equation S8) because we find that this substantially improves full variation performance. Most of the variation in nightlight luminosity occurs within countries, rather than between countries, which is considerably different from what we observe for HDI and IWI. Importantly, the choice to use a different

re-centering procedure for NL does not impact the downscaled within-province performance (Figure 2J), which we believe provides the most important evaluation of downscaling performance.

Supplementary References

[67] European Union Copernicus. Harmonized Sentinel-2 MSI: Multispectral Instrument, Level-2A (SR). URL https://developers.google.com/earth-engine/datasets/catalog/COPERNICUS_S2_SR_HARMONIZED. (2023).

[68] Norström, A. V. *et al.* Principles for knowledge co-production in sustainability research. *Nature sustainability* **3**, 182–190 (2020).

[69] Kliskey, A. *et al.* Building trust, building futures: Knowledge co-production as relationship, design, and process in transdisciplinary science. *Frontiers in Environmental Science* **11**, 137 (2023).

[70] Microsoft. Bing Maps - Global ML Building Footprints. URL https://github.com/microsoft/GlobalMLBuildingFootprints/tree/main?tab=readme-ov-file. (2022).

[71] Open Street Maps. API. URL https://wiki.openstreetmap.org/wiki/API_v0.6. (2021).

[72] Shaver, A., Carter, D. B. & Shawa, T. W. Terrain ruggedness and land cover: Improved data for most research designs. *Conflict Management and Peace Science* **36**, 191–218 (2019).

[73] Hansen, M. C. *et al.* High-resolution global maps of 21st-century forest cover change. *Science (New York, N.Y.)* **342**, 850–3 (2013).

[74] Ramankutty, N., Evan, A. T., Monfreda, C. & Foley, J. A. Farming the planet: 1. geographic distribution of global agricultural lands in the year 2000. *Global Biogeochemical Cycles* **22** (2008).

[75] EarthStat. Cropland and Pasture Area in 2000. URL http://www.earthstat.org/. (2008).

[76] NOAA Physical Sciences Laboratory. CPC Global Unified Temperature. URL https://psl.noaa.gov/data/gridded/data.cpc.globaltemp.html. (2024).

[77] NASA. Global Precipitation Measurement. IMERG Grand Average Precipitation Climatology. URL `https://gpm.nasa.gov/data/imerg/precipitation-climatology`. (2024).

[78] Pokhriyal, N. & Jacques, D. C. Combining disparate data sources for improved poverty prediction and mapping. *Proceedings of the National Academy of Sciences* **114**, E9783–E9792 (2017).
